# Community-level Social Vulnerability and Cervical Cancer Mortality Among Young and Old Adults in the State of Alabama

**DOI:** 10.1007/s10900-025-01482-6

**Published:** 2025-06-01

**Authors:** Pranali G. Patel, Sabrina Chowdhury, Howard W. Wiener, Justin T. George, Ehsan Abdalla, Yuanfan Ye, Teresa K. L. Boitano, Staci L. Sudenga, Gabriela R. Oates, Sadeep Shrestha

**Affiliations:** 1 Department of Epidemiology, School of Public Health, University of Alabama at Birmingham, Birmingham, AL USA; 2 Alabama Department of Public Health, Birmingham, AL USA; 3 Department of Graduate Public Health, Tuskegee University, Tuskegee, AL USA; 4 Department of Obstetrics and Gynecology, Heersink School of Medicine, University of Alabama at Birmingham, Birmingham, AL USA; 5 Division of Epidemiology, Vanderbilt University Medical Center, Nashville, TN USA; 6 Department of Medicine, Heersink School of Medicine, University of Alabama at Birmingham, Birmingham, AL USA; 7 RPHB 217L, Ryals Public Health Building (RPHB), 1665 University Boulevard, 35242 Birmingham, AL USA

**Keywords:** Cervical cancer, HPV, Mortality, Socioeconomic, Community-level Social Vulnerability

## Abstract

In addition to individual factors, differences in community-level factors impact mortality rates of cervical cancer (CC), especially in the Southeast United States, where CC one-year mortality is significantly higher than national average. This study investigated the association between community-level social vulnerability measured using the Centers for Disease Control and Prevention’s Social Vulnerability Index (SVI) and overall and one-year CC mortality in Alabama. Retrospective cohort study using Alabama State Cancer Registry data from 2012 to 2021. Outcome of interest was mortality due to CC. Residential addresses were geocoded to determine SVI scores categorized into quartiles. Cox proportional hazards model was used to assess associations between SVI quartiles and overall and one-year CC mortality adjusting for age at diagnosis, race, marital status, and insurance status. Further, CC mortality in younger adults (≤50 years) was compared with older adults (> 50 years). A total of 1,325 women with CC were included in the study. The median age at diagnosis was 49 years (IQR: 39–62) and 69.73% were White. Median follow-up time was 9 months (IQR: 5–17). Among older adults, we observed statistically significant association between higher SVI quartiles and overall mortality (Q4: aHR 1.86; 95% CI 1.15, 3.01; *p* = 0.012] and one-year mortality (Q3: aHR 2.66; 95% CI 1.34, 5.29; *p* = 0.005; Q4: aHR 2.45; 95% CI 1.18, 5.08; *p* = 0.016). This study highlights the role of community factors in CC mortality among older women. Community-level strategies are needed to reduce the burden of CC mortality in Alabama and other high-risk regions.

## Introduction

Cervical cancer remains a significant public health issue and is the fourth cause of cancer morbidity and mortality among women worldwide [16]. About 90% of cervical cancer cases occur due to human papillomavirus (HPV) [5]. Although cervical cancer is preventable through HPV vaccination and screening, an estimated 13,820 new cases are diagnosed and 4,360 women die from the disease each year in the United States (U.S.) [23]. Alabama has one of the highest incidence and mortality rate of HPV-associated cancers in the U.S. and often ranks among the lowest in various health metrics [4], with significant disparities in health outcomes by age, race, ethnicity, and socioeconomic status (SES). In 2021, the cervical cancer incidence rate was 9.5 per 100,000 in Alabama, one of the highest in the country, and the cervical cancer mortality rate was higher than the national average (3.1 vs. 2 per 100,000) [27]. Additionally, non-Hispanic Black women have higher incidence (8.1 vs. 6.8 per 100,000) and mortality (2.7 vs. 2.0 per 100,000) rates of cervical cancer compared to non-Hispanic White women [27], with disparities worsening in low-income counties [3].

Community-level factors, such as neighborhood social, economic, and living conditions, contribute to outcomes in cervical cancer. One measure of such community conditions is the Social Vulnerability Index (SVI) developed by the Centers for Disease Control and Prevention (CDC) [28]. The index is a composite measure of community vulnerability calculated from variables collected by the American Community Survey. It allows for an assessment of the influence of community-level factors on cancer across neighborhoods and geographic regions [26]. Prior studies have found SVI to be associated with incidence and mortality in other cancers [15, 17, 18]. A study of 3,141 U.S. counties reported that higher SVI is significantly associated with lower cervical cancer screening rates [6], implying an impact on cancer incidence and mortality. Similarly, a review of 31 studies emphasized SVI’s role in identifying disparities in cancer patient outcomes [26]. However, studies on the role of SVI in cervical cancer mortality remain limited.

Given the higher burden of cervical cancer in Alabama, a comprehensive understanding of the role of community environments is crucial. To address this gap, we determined the one-year and overall survival of cervical cancer patients and the independent effect of SVI on survival rates utilizing data from the Alabama Statewide Cancer Registry (ASCR). In addition, we explored the association between SVI and cervical cancer mortality among younger and older adult cancer patients.

## Methods

### Study Design and Setting

We conducted a retrospective cohort study using ASCR data from 2012 to 2021. The ASCR is a comprehensive, population-based cancer registry established in 1995 by the Alabama Department of Public Health [2]. The registry systematically collects data on all cancer cases diagnosed or treated in Alabama, thereby monitoring trends in cancer incidence, identifying individuals at high risk for cancer. All procedures handling and analyzing ASCR data have been reviewed and approved by the University of Alabama at Birmingham (UAB) Institutional Review Board (IRB). For this study, we included patients with confirmed diagnosis of cervical cancer identified using the International Classification of Diseases, Tenth Revision (ICD-10) code C53 (C53.0, C53.1, C53.8, and C53.9). We excluded women with missing residential addresses.

### Exposure

Our primary exposure variable was the 2010 SVI, a composite measure calculated from 14 variables collected annually by the American Community Survey and aggregated to Census tracts using 5-year estimates. The index provides an overall vulnerability score and four thematic subscores of a community’s (1) socioeconomic status, (2) household composition and disability, (3) minority status and language, and (4) housing type and transportation [28]. Patient residential census tracts available in the ASCR were linked to SVI scores using 11-digit Federal Information Processing Standard (FIPS) codes. The SVI uses percentile ranking (0 to 1), with higher values indicating greater vulnerability. For this analysis, SVI overall and thematic scores were categorized into quartiles based on the distribution within the study population (lowest quartile = least vulnerable, highest quartile = most vulnerable).

### Outcome

Our primary outcome of interest was cervical cancer mortality. Individuals who were alive or lost to follow-up at last contact were censored. This information was based on vital status data obtained from the ASCR, which includes comprehensive data from medical records, death certificates, reports from healthcare providers, and cancer registry entries [13]. Follow-up time was defined as the date of the last follow-up or death from the date of cervical cancer diagnosis.

### Covariates

Covariates were examined based on their relevance to cervical cancer outcomes in the literature and their potential to confound the relationship between area-level social vulnerability and cervical cancer mortality. Known confounders, age at diagnosis (in years as continuous variable), self-reported race (Black/White), type of insurance (Insured/Private Insurance/Medicare/Medicaid), and marital status (Single/Married/Divorced) were adjusted in all analyses [20, 32].

### Statistical Analysis

We used descriptive statistics to compare participant characteristics overall and by SVI quartiles. We reported frequency and percentages for categorical variables and median and interquartile range (IQR) for continuous variables. We used Cox proportional hazards models to estimate hazard ratios (HR) and 95% confidence intervals (CI) to assess the association between overall SVI (quartiles) and cervical cancer mortality. Cox regression models were adjusted for age at diagnosis (as continuous), race, marital status, and insurance type. The proportional hazards assumption was tested and confirmed for all the covariates. Since our data showed a higher proportion of cervical cancer mortality in the first year, we specifically assessed the association between overall SVI and one-year mortality. We performed a stratified analysis to examine whether the association between overall SVI and cervical cancer mortality varied among older (> 50 years) and younger adults (≤50 years), as defined by the National Cancer Institute (NCI) [23]. We adjusted for age within each group to account for the range of age distribution. For all mortality associations observed with overall SVI score, we further examined the association with each of the four themes of SVI. We plotted a Kaplan-Meier survival curve to show the differences in survival by time-at-risk (in months) and estimated survival probabilities for overall and at one year stratified by SVI quartile groups (lowest vs. highest) in older women. Statistical significance was set at *p* < 0.05. All statistical analyses were performed using SAS software, version 9.4 (SAS Institute Inc., Cary, NC) and R version 4.5.0 (2025-04 ID="MN1">−11).

## Results

A total of 1,325 women with cervical cancer were included in the study. The median age at diagnosis was 49 years (IQR: 39–62 years). The majority (*n* = 903, 69.7%) were White, 484 (36.5%) had private insurance, and 510 (42.1%) were married. During the study period, 338 (26.0%) women died from cervical cancer, of whom 170 (50.2%) died in the first year. The median follow-up time since cervical cancer diagnosis was 9 months (IQR: 5–17 months). We observed a similar median age at diagnosis across the SVI quartiles. However, an increase in the number of Black women, women without insurance, and single women was observed in communities with higher SVI scores (Table 1).

**Table 1:** Baseline characteristics of participants, overall and by Social Vulnerability Index (SVI) quartiles

Characteristics	OverallN (%)^#^	Social Vulnerability Index (SVI) Quartiles*			
		Q1N (%)^#^	Q2N (%)^#^	Q3N (%)^#^	Q4N (%)^#^
Age at cancer diagnosis (in years), median (IQR)	49 (39–62)	47 (39–61)	49 (40–62)	51 (39–64)	50 (40–61)
*Race*					
White	903 (69.73)	277 (86.83)	258 (78.42)	233 (71.25)	135 (42.19)
Black	392 (30.27)	42 (13.17)	71 (21.58)	94 (28.75)	185 (57.81)
*Insurance*					
Not insured	124 (9.36)	25 (7.55)	30 (9.06)	32 (9.67)	37 (11.14)
Private insurance	484 (36.53)	168 (50.76)	130 (39.27)	107 (32.33)	79 (23.80)
Medicaid	381 (28.75)	61 (18.43)	95 (28.70)	106 (32.02)	119 (35.84)
Medicare	336 (25.36)	77 (23.26)	76 (22.96)	86 (25.98)	97 (29.22)
*Marital Status*					
Single	351 (27.42)	51 (16.35)	88 (28.85)	78 (26.71)	134 (44.37)
Married	510 (42.11)	169 (54.17)	129 (42.30)	129 (44.18)	83 (27.48)
Divorced/Separated/Widowed	350 (28.90)	92 (29.49)	88 (28.85)	85 (29.11)	85 (28.15)

^#^N (%) – frequency and column percentages

*SVI quartiles – 0 < Q1 ≤ 0.311, 0.311 < Q2 ≤ 0.532, 0.532 < Q3 ≤ 0.770, Q4 > 0.770

Only complete observations were included in the analyses

Table 2 shows the association between SVI quartiles and overall cervical cancer mortality. In the unadjusted analysis, we observed an increase in hazard of mortality from SVI Q2 to Q4 compared to Q1, but the association was not statistically significant. The association did not reach statistical significance also after adjusting for age at diagnosis, race, marital status, and insurance type.

**Table 2:** Association between Social Vulnerability Index (SVI) and Cervical Cancer Mortality (N: overall = 1168; one year = 751)

Social Vulnerability Index (SVI)	Crude HR (95% CI)	*p*-value	Adjusted HR (95% CI)^#^	*p*-value
*Overall Mortality*				
Q1	Ref		Ref	
Q2	1.03 (0.75, 1,41)	0.873	0.99 (0.70, 1.38)	0.929
Q3	1.06 (0.76, 1.46)	0.743	1.05 (0.74, 1.48)	0.790
Q4	1.31 (0.97, 1.77)	0.078	1.27 (0.90, 1.78)	0.168
*One year Mortality*				
Q1	Ref		Ref	
Q2	1.45 (0.90, 2.34)	0.130	1.27 (0.76, 2.13)	0.361
Q3	1.59 (0.99, 2.55)	0.054	1.58 (0.96, 2.61)	0.075
Q4	1.95 (1.24, 3.07)	0.004^*^	1.66 (0.99, 2.80)	0.056

HR – Hazard Ratio; 95% CI – Confidence interval

Cox model was used to examine association between SVI and cervical cancer mortality

^#^Adjusted for age, race, marital status, and insurance type

Ref – Reference; p-value was set at < 0.05

Only complete observations were included in the analyses

*SVI quartiles – 0 < Q1 ≤ 0.311, 0.311 < Q2 ≤ 0.532, 0.532 < Q3 ≤ 0.770, Q4 > 0.770

Table 3 shows the age-specific subgroup analysis. In younger women (≤ 50 years), we did not find a statistically significant association between overall SVI and cervical cancer mortality in both unadjusted and adjusted models. However, in older women (> 50 years), the unadjusted model showed a 60% increased hazard of mortality in overall SVI Q4 (HR: 1.60; 95% CI: 1.04, 2.47; *p* = 0.033) compared to Q1. In the adjusted model, this hazard increased to 86% (adjusted hazard ratio (aHR): 1.86; 95% CI: 1.15, 3.01; *p* = 0.012).

**Table 3:** Association between Social Vulnerability Index (SVI) and Cervical Cancer Mortality among Women Younger (N: overall = 600; one year = 417) andOlder than 50 years (N: overall = 568; one year = 334)

Social Vulnerability Index (SVI)	≤50 years				> 50 years			
	Crude HR (95% CI)	p-value	Adjusted HR (95% CI) ^*#*^	p-value	Crude HR (95% CI)	p-value	Adjusted HR (95% CI) ^*#*^	p-value
*Overall Mortality*								
Q1	Ref		Ref		Ref		Ref	
Q2	0.78 (0.50, 1.22)	0.281	0.69 (0.43, 1.11)	0.128	1.31 (0.82, 2.08)	0.256	1.31 (0.80, 2.12)	0.284
Q3	0.75 (0.45, 1.23)	0.252	0.63 (0.36, 1.09)	0.096	1.37 (0.88, 2.14)	0.164	1.51 (0.95, 2.42)	0.083
Q4	1.04 (0.68, 1.59)	0.852	0.80 (0.48, 1.32)	0.381	1.60 (1.04, 2.47)	0.033^*^	1.86 (1.15, 3.01)	0.012^*^
*One year Mortality*								
Q1	Ref		Ref		Ref		Ref	
Q2	1.06 (0.53, 2.16)	0.864	0.77 (0.35, 1.65)	0.497	1.80 (0.92, 3.50)	0.084	1.79 (0.86, 3.73)	0.118
Q3	0.76 (0.34, 1.69)	0.495	0.64 (0.26, 1.58)	0.335	2.24 (1.19, 4.24)	0.013^*^	2.66 (1.34, 5.29)	0.005^*^
Q4	1.60 (0.83, 3.10)	0.159	0.98 (0.44, 2.19)	0.960	2.27 (1.19, 4.30)	0.013^*^	2.45 (1.18, 5.08)	0.016^*^

HR – Hazard Ratio; 95% CI – Confidence interval

Cox model was used to examine association between SVI and cervical cancer mortality

^#^Adjusted for age, race, marital status, and insurance type

Ref – Reference; p-value was set at < 0.05

Only complete observations were included in the analyses

*SVI quartiles – 0 < Q1 ≤ 0.311, 0.311 < Q2 ≤ 0.532, 0.532 < Q3 ≤ 0.770, Q4 > 0.770

Furthermore, we observed an increased hazard of one-year mortality with higher overall SVI scores. In the unadjusted model, women residing in SVI Q4 areas had a 95% significantly higher hazard of mortality within one-year of diagnosis compared to counterparts in SVI Q1 areas (HR: 1.95; 95% CI: 1.24, 3.07; *p* = 0.004). However, after adjusting for covariates, the association between SVI quartiles and one-year mortality was no longer statistically significant **(**Table 2**).**

When stratified by age group, the association between SVI quartiles and one-year mortality in younger women was not statistically significant. However, among older women, the unadjusted model showed a statistically significant increase in hazard of one-year mortality in SVI Q3 (HR: 2.24; 95% CI: 1.19, 4.24; *p* = 0.013) and SVI Q4 (HR: 2.27; 95% CI: 1.19, 4.30; *p* = 0.013) compared to SVI Q1. The association remained statistically significant after adjusting for covariates, with increased one-year mortality observed in SVI Q3 (aHR: 2.66; 95% CI: 1.34, 5.29; *p* = 0.005) and SVI Q4 (aHR: 2.45; 95% CI: 1.18, 5.08; *p* = 0.016) compared to SVI Q1 **(**Table 3**)**.

**Fig. 1:**
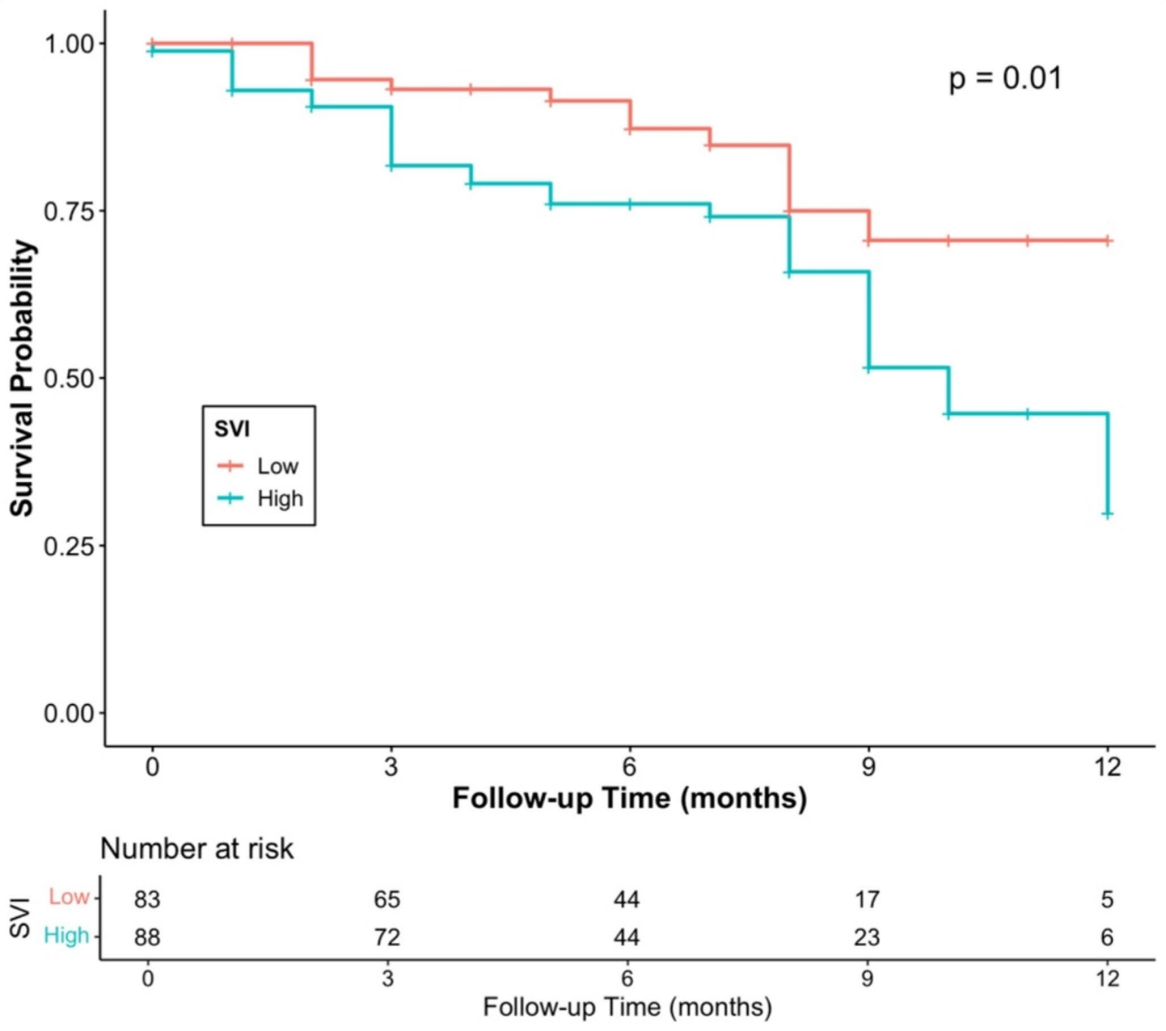
Kaplan-Meier Curve for One Year Mortality by SVI quartile (highest vs. lowest) in Older Women (> 50 years) with Cervical Cancer (*N* = 171)

**Fig. 2:**
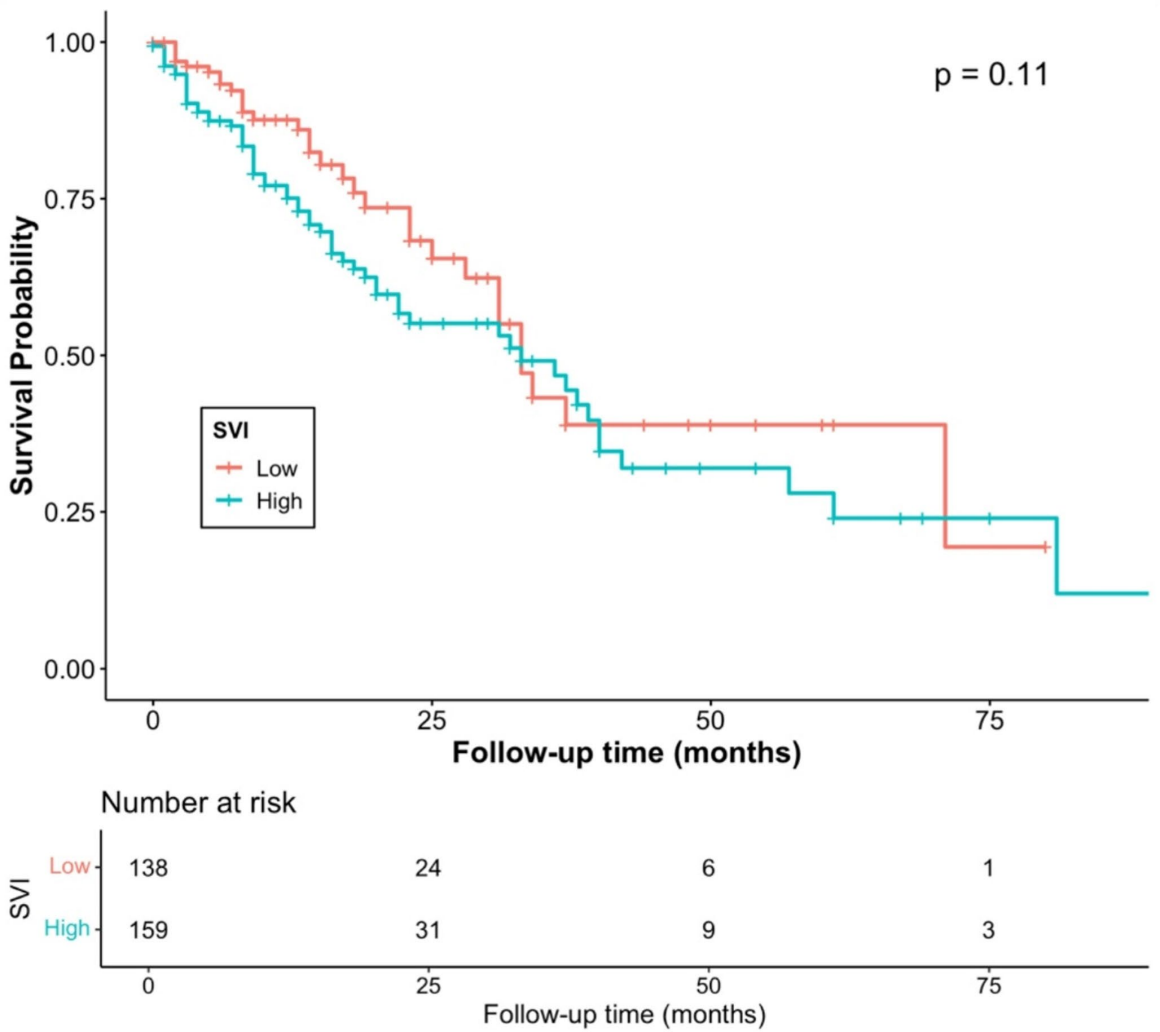
Kaplan-Meier Curve for Overall Mortality by SVI Quartile (highest vs. lowest) in Older Women (> 50 years) with Cervical Cancer (*N* = 297)

Figure 1 represents the Kaplan-Meier survival curve for one-year mortality among older women, showing that women with cervical cancer residing in vulnerable communities (SVI Q4) have lower survival probabilities compared to counterparts residing in less vulnerable areas (SVI Q1). Figure 2 shows the Kaplan-Meier survival curve for overall mortality among older women, showing no significant differences in the survival probabilities between the highest (Q4) and the lowest (Q1) SVI groups. Finally, in the sensitivity analysis, SVI Theme 2 (household composition and disability) accounted for the increase in both overall Q4 (aHR: 1.64; 95% CI: 1.04, 2.56; *p* = 0.032) and one-year Q2 (aHR: 1.99; 95% CI: 1.02, 3.87; *p* = 0.042); Q3 (aHR: 2.30; 95% CI: 1.18, 4.47; *p* = 0.015); Q4 (aHR: 2.58; 95% CI: 1.30, 5.15; *p* = 0.007) mortality among older women.

## Discussion

The present study is one of the first to examine the relationship between community- level factors measured with the CDC’s SVI and cervical cancer mortality in Alabama using the state registry data. Our findings provide information on community-level social vulnerability and age-related disparities in cervical cancer mortality. Although not significant in the overall study population, our results suggest an increasing trend in hazard of mortality with increased community-level social vulnerability after adjusting for age, race, marital status, and insurance type. This association was significantly stronger in magnitude among older women, with nearly double hazard in mortality in the highest vulnerability group compared to the lowest. Specifically, older women who lived in areas with high overall vulnerability showed increased risk of cervical cancer mortality within one year. These observations highlight community-level disparities that warrants further investigation.

Our finding aligns with other studies linking community vulnerability with poorer cancer outcomes. A study conducted in Ohio reported a higher cervical cancer mortality rate in high SVI counties (2.5 vs. 1.8 per 100,000) compared to low SVI counties [24], emphasizing the impact of social factors on cancer outcomes. Similar findings have been observed at the regional and national levels for lung, colorectal, pancreatic, breast, and prostate cancer, with increased incidence and mortality rates in high SVI counties of Southeastern states [18, 29]. In Alabama, despite increased screening rates, cervical cancer mortality remains disproportionately high, specifically among Black compared to White women [1, 11, 31]. However, in race-specific subgroup analysis, we did not observe significant differences between Black and White women in the association between SVI and cervical cancer mortality in our study (data not shown).

Our study findings are consistent with previous literature that reported declining survival with increasing age among women with cervical cancer [10, 14, 21]. Studies have suggested that factors such as socioeconomic status [9, 14, 19], pre-existing comorbidities [22], advanced cancer stage [10], and health literacy [12, 25] may explain the increased mortality observed in older women. While comorbidities in older women may contribute to poorer overall survival [30], the strong association observed between SVI Theme 2 (household composition and disability) and cervical cancer mortality among older women suggests that those living in communities with a greater proportion of vulnerable residents (individuals aged 65 years and older, 17 years and younger, civilians with a disability, single-parent households, and individuals with limited English language proficiency) may face additional risks. These factors may contribute to untimely screenings, non-adherence to treatment, and comorbidities, further leading to increased mortality rate.

Our study is one of the first to report an association between community-level social vulnerability and one-year mortality in women with cervical cancer, which may reflect the effect of pre-existing health conditions, inadequate access to healthcare, and low socioeconomic status on survival outcomes. One-year mortality is specifically relevant in cervical cancer, as any delays in early diagnosis and treatment initiation can significantly affect survival. Few other studies have shown results similar to our findings that women in areas with higher social vulnerability were more likely to experience increased cancer-specific mortality [7, 18]. Our study documents disparities in cervical cancer mortality among the older population [8]. These findings highlight the need for integrated care approaches that address both existing comorbidities and cancer-specific treatment, which may help reduce mortality.

As with most analyses of state registry data, our study has limitations. Firstly, approximately half (47.9%) of the patients in the registry had missing data for a stage of cancer. Future studies should explore the association between SVI and cervical cancer mortality across stages of cancer. Secondly, we did not have information on treatment initiation, treatment adherence, or pre-existing comorbid conditions, which limited our understanding of the role of these factors in mortality outcomes. Lastly, the study is not generalizable to other regions of the U.S.

Despite the limitations, our study provides insights into the association of community-level social vulnerability and cervical cancer mortality rate in Alabama, with worse outcomes observed in older women. The study has important implications for public health strategies aimed at reducing the burden of cervical cancer mortality in high-risk regions. Additionally, it is important to distinguish between younger and older cancer patients as they face different healthcare challenges and access, comorbidity burden, and screening practices. Future analyses should focus on larger, nationally representative samples that account for comorbidity burden and staging of cancer. Interventions should address vulnerable populations such as older women in order to mitigate the burden of cervical cancer and reduce disparities among population groups.

## Data Availability

The deidentified data from the Alabama Statewide Cancer Registry (ASCR) that support the findings of this study are available from the corresponding author upon reasonable request, following the institutional data sharing agreement.
